# Lower extremity compression garments use by athletes: why, how often, and perceived benefit

**DOI:** 10.1186/s13102-020-00230-8

**Published:** 2021-03-24

**Authors:** Thierry P. C. Franke, Frank J. G. Backx, Bionka M. A. Huisstede

**Affiliations:** Department of Rehabilitation, Physical Therapy Science & Sport, Brain Center, University Medical Center Utrecht, Utrecht University, Utrecht, The Netherlands

**Keywords:** Stockings, compression [MeSH], Athletes* [MeSH], Sports injuries [MeSH], Epidemiology [MeSH]

## Abstract

**Background:**

Studies on the benefits of lower extremity compression garments (CGs) have focused on their effects on post-exercise recovery and performance improvement. Less is known about why athletes actually use CGs, the frequency with which they use them, and perceived benefits from using CGs. The purpose of this study was to investigate which athletes use CGs, why athletes use CGs, when CGs are worn by athletes, and, in case of an injury or injury prevention, for which injuries CGs are used.

**Methods:**

This cross-sectional study involved 512 athletes who used lower extremity CGs. Athletes completed a questionnaire on the type of CGs they used, and when and why they used them. They also reported their sports participation, past and current sports injuries, and the perceived benefits of using CGs.

**Results:**

88.1% (*n*=451) of the athletes were endurance athletes and 11.9% (*n*=61) were non-endurance athletes. Endurance and non-endurance athletes reported running (84.7%, *n*=382) and obstacle course racing (24.6%, *n*=15) the most frequently as primary sports, respectively. The most-used CG was the compression sock (59.2%, *n*=303). In total, 47.5% (*n*=246) of the athletes used a CG primarily to prevent re-injury and 14.5% (*n*=74) to reduce symptoms of a current sports injury. Other primary reported aims were primary prevention (13.6%), post-exercise recovery (14.3%), sports performance improvement (8.8%), and to look good (0.2%). The point prevalence of past and current sports injuries among all athletes was 84.2 and 20.2%, respectively. The most common current sports injuries were shin and calf injuries. Many athletes “always” or “often” used their CGs during training (56.8%, *n*=279) and competitions (72.9%, *n*=264). Furthermore, almost 90% of the athletes that aimed to prevent re-injury by using CGs reported that CGs contributed to secondary injury prevention.

**Conclusion:**

88% of the CG-users were endurance athletes, of which 85% were runners. All athletes mainly used CGs to prevent injury recurrence, but also to reduce symptoms of a current sports injury. A majority of the athletes reported positive perceived effects from the CGs. CGs were used more during than after sports participation.

## Key points

- CGs were mostly used by endurance athletes, of which more than 80% were runners.
- Over 80% of all athletes aimed to prevent re-injury by using CGs; for almost 50% of these athletes secondary sports injury prevention is the most important/primary reason for wearing CGs.
- Other primary reasons indicated for the use of CGs were reducing symptoms of a current sports injury (14.5%), post-exercise recovery (14.3%), primary prevention (13.6%), and sports performance improvement (8.8%).
- Almost 90% of those aimed to reduce recurrent injuries, reported perceived effects of the use of CGs with regard to this goal/purpose.
- Of those indicating that they use CGs for recovery after sports or improvement of sports performance, over 80% perceived faster recovery and over 70% perceived sports performance improvement respectively.
- Compression garments are used more during than after sports participation.
- For runners, always using their compression sock or tube during training and competition and an average running distance of ≥23 km was significantly associated with a lower prevalence of lower extremity sports injuries.

## Background

The popularity of lower extremity compression garments (CGs) amongst athletes continues to increase [1, 2]. Initially CGs were mainly prescribed to patients with chronic venous disorder [3]. Using graduated lower extremity CGs with a degrading pressure from distal to proximal parts of this extremity increases venous flow velocity, reduces venous wall distension, improves valvular function, and stimulates lymphatic outflow [3]. Consequently, using graduated CGs diminishes venous hypertension and symptoms of the swollen extremity and improves venous hemodynamics of the affected extremity [3, 4]. Ultimately, the degrading pressure helps the venous blood to return to the heart [5]. Since Berry and McMurray [6] originally investigated the effects of CGs for athletes, research on their effects on sports performance and post-exercise recovery keeps emerging and the popularity of CG keeps growing among athletes.

Multiple meta-analyses have investigated the effects of CGs on sports performance and post-exercise recovery [7–9]. Engel et al. [7] found in their meta-analysis that using compression clothing, defined as knee-high socks, sleeves, or shorts, slightly improves running economy (i.e. the energy expenditure at a submaximal velocity, expressed using oxygen consumption [10]) (mean Hedges G = 0.21±0.38; range 0.00–0.88). Moreover, they reported that using compression sleeves or stockings slightly improves biomechanical variables (mean G= 0.21±0.38; range − 0.33 to 0.72), such as ground contact time, step frequency, step length and swing time, and the psychological variable perceived exertion (mean G= 0.28±0.38; range − 0.31 to 1.21) [7]. Whereas, the meta-analysis by Da Silva et al. [9] found no effect of lower leg CGs on high-intensity exercise performance, defined as time difference in a maximum running test across specific distances (50–400 m, 800–3000 m, or > 5000 m), compared to no CGs or placebo garments. Regarding post-exercise recovery, two meta-analyses showed that using CGs decreases post-exercise leg soreness and delayed the onset of muscle fatigue and exercise induced-muscle damage respectively [7, 8]. These effects were attributed to an enhanced venous blood flow and lymphatic outflow [7, 8]. Unfortunately, the aforementioned meta-analyses have included studies of different types of CGs, with varying pressure gradients, and different populations, which could have introduced bias into their analyses. Thus, the literature remains inconclusive on the physiological effects of CGs. Nonetheless, in clinical practice sports medicine physicians, physiotherapists, and manufacturers suggest that CGs can be used to prevent sports injuries or to reduce symptoms of a current sports injury [11, 12]. However, in scientific literature no information can be found on the effect of CGs on the prevention of (recurrent) sports injuries. Thus, there is a gap between the clinical perspective on the application of CGs and the scientific literature as it is unclear why CGs are used to prevent or treat sports injuries. Moreover, little is reported in the scientific literature about the type of athletes that use CGs, for which purposes they use CGs, and whether they perceive any effects from using CGs. This information can give direction to future (epidemiologic) research on this topic. Furthermore, it could inform clinicians and researchers on the expectations of athletes using CGs. Moreover, if athletes report using CGs for sports injury prevention or to reduce symptoms of a current sports injury, it would be of interest to know for which type of injury [13, 14]. Therefore, the aim of this study was to investigate which athletes use CGs, why athletes use CGs, when CGs are worn by athletes, and, in case of an injury or injury prevention, for which injuries CGs are used. Moreover, we investigated the perceived effects of the use of CGs by the athletes on primary and secondary injury prevention, symptoms from current sports injuries, post-exercise recovery and sports performance. Additionally, we studied the association between the characteristics of the athletes, the use of CGs and the odds of a current lower leg sports injury.

## Method

### Design

This cross-sectional study was approved by the University Medical Center Utrecht ethics committee (protocol number 16–781/C). The manuscript was written using the Strengthening the Reporting of Observational Studies in Epidemiology (STROBE) guideline [15].

### Participants

771 adult athletes (≥18 years), with adequate Dutch language skills, who used lower extremity CGs from ‘Herzog Medical’ (Herzog Medical B.V. Woudenberg, the Netherlands), regardless of the level in which they participated in their sports, were eligible for inclusion in our study. We chose to include athletes using CGs from only one manufacturer to ensure that the garments had similar weaves and pressure gradients.

### Procedures

The athletes were recruited in April 2017. First, the manufacturer asked the athletes if they were interested in participating in this study. If so, the researchers continued the study, independently of the manufacturer. Each interested athlete received an information letter with details of the study from the researchers and was invited to participate. Four weeks later, athletes who were willing to participate, received an e-mail containing a hyperlink, which could be used to access the questionnaire in NetQ (NetQuestionnaires, NetQ Healthcare B.V., Amsterdam, The Netherlands). The athletes could only fill in the questionnaire after they provided written informed consent. They had five weeks to fill in the questionnaire. Athletes who had not completed or started the questionnaire were sent a reminder every two weeks.

### Questionnaire

#### Characteristics of the athletes

Athletes provided information about their age, sex, body mass, height, and, where appropriate, comorbidities. They also provided information about the sports they practised, training frequency, and participation in competitions in the three months before study inclusion. The three-month period was chosen in order to reduce the risk of recall bias.

For this study athletes were divided into two sports categories: endurance and non-endurance athletes (Table 1). This categorization was adapted from previous studies [16, 17]. Endurance sports are characterized by repeated contractions of large skeletal muscle groups at a submaximal intensity over prolonged periods of time for which the energy is delivered mainly by the aerobic system [18]. The main purpose of endurance sports is to increase endurance performance, i.e. to progressively increase the anaerobic threshold (i.e. the start of anaerobic metabolism towards higher exercise intensity). For the purpose of this study, non-endurance athletes were those athletes who did not report an endurance sport as their primary sport. Questions included in the section on the characteristics of the athletes were adapted from previously published studies [19–21].

**Table 1:** Sample characteristics

	Endurance athletes (***n*** = 451)		Non-endurance athletes (***n*** = 61)		***P***-value
Basic characteristics					
Sex, Female/Male, n (%)	255/196	(56.5/43.5)	39/22	(63.9/36.1)	0.273^*^
Age years, median (IQR)	41	(34.0-48.0)	34.0	(23.5-42.0)	0.739^†^
Height, cm, mean (SD)	177.6	(8.6)	174.8	(8.9)	0.756^‡^
Body mass, kg, median (IQR)	71.0	(63.0-80.0)	72.0	(63.5-79.5)	0.782^†^
Primary sport, n (%)					
Running	382	(84.7)	-		
Trail running	5	(1.1)	-		
Road cycling/ mountain biking/ tour cycling	24	(5.3)	-		
Triathlon	27	(6.0)	-		
(Nordic) Walking/ racewalking	6	(1.3)	-		
Canicross	5	(1.0)	-		
Spinning	1	(0.2)	-		
Speed skating	1	(0.2)	-		
Soccer	-		2	(3.2)	
Hockey	-		3	(4.9)	
Korfball	-		3	(4.9)	
Basketball	-		1	(1.6)	
Handball	-		1	(1.6)	
Athletics	-		6	(9.8)	
Tennis	-		8	(13.1)	
Volleyball	-		4	(6.6)	
Fitness (cardio and strength training)	-		10	(16.4)	
Obstacle course racing / survival running	-		15	(24.6)	
Bootcamp	-		2	(3.2)	
Kickboxing	-		3	(4.9)	
Other	1	(0.2)	3	(4.9)	
Number of sports in which athlete participated, n (%)					
1 sport	105	(23.6)	10	(16.4)	
2 sports	199	(44.7)	22	(36.1)	
3 sports	141	(31.7)	27	(44.3)	
Primary sport					
Average training load/week during the last 3-months, hours, median (IQR)	4.0	(2.8-5.0)	3.0	(3.0-5.0)	0.900^†^
Could not train for primary sport because of sports injury, n (%)	10	(2.2)	1	(1.7)	
Competition participation during the last 12 months, median (IQR)	12.0	(5.0-24.0)	30	(12.0-52.0)	**<0.001**^†^
Secondary sport					
Average training load/week during the last 3 months, hours, median (IQR)	2.0	(1.0-3.0)	2.0	(2.0-3.0)	0.090^†^
Could not train for secondary sport because of sports injury	11	(3.2)	1	(2.0)	
Competition participation during the last 12 months, median (IQR)	12.0	(2.0-12.0)	12.0	(3.2-21.0)	0.462^†^
Tertiary sport					
Average training load/week during the last 3 months, hours, median (IQR)	1.0	(1.0-3.0)	2.0	(1.0-3.0)	0.557^†^
Could not train for secondary sport because of sports injury	0	(0)	0	(0)	
Competition participation during the last 12 months, median (IQR)	7.0	(2.0-18.0)	8.0	(3.5-12.0)	0.890^†^
Total^§^ average training load/week during the last 3 months, hours, median (IQR)	5.0	(4.0-8.0)	6.0	(5.0-10.0)	**0.038**^†^
Total^§^ competition participation during the last 12 months, median (IQR)	12.0	(6.0-24.0)	36.0	(12.0-52.0)	**<0.001**^†^
Average running distance per week during the last 3 months for athletes whose primary sport was running, kilometres (*n* = 381), n (%)					
0-5	20	(5.2)	-		
6-10	39	(10.2)	-		
11-22	109	(28.6)	-		
23-42	150	(39.4)	-		
>42	63	(16.5)	-		
Type of CGs, n (%)					
PRO sports compression sock	259	(57.4)	44	(72.1)	
PRO sports compression tube	127	(28.2)	11	(18.0)	
Herzog ankle compression socks	34	(7.5)	4	(6.6)	
Active compression garments	24	(5.3)	2	(3.3)	
Thigh support (hamstring garment)	2	(0.4)	0	(0.0)	
PRO compressive knee support garment	2	(0.4)	0	(0.0)	
Other Herzog compression garment	3	(0.7)	0	(0.0)	

*Abbreviation: SD* standard deviation, *IQR* 25 to 75% inter-quartile range

^*^Tested using a chi-squared test

^†^Tested using Mann-Whitney U-test

^‡^tested using t-test

^§^Total regards the sum of the primary, secondary, and tertiary sports

#### Sports injuries

If athletes reported having a past or current lower extremity sports injury, they were asked about its onset mechanism (i.e. acute or gradual), whether they used their CG for this injury, time since occurrence, and the location, type, and duration of the injury. A sports injury was defined as any self-reported physical complaint deemed by the athletes themselves to be caused by participating in their sport and which rendered the athlete unable to participate (fully) in their sport(s), irrespective of the need for medical attention [22].

#### Compression garments (CGs)

The athletes reported which type of CG they used: PRO sports compression socks (pressure; about 30 mmHg around the ankle and about 23 mmHg around the knee), PRO sports compression tubes (pressure; about 30 mmHg of pressure around the ankle and about 23 mmHg of pressure around the knee), active compression garments (pressure; about 28 mmHg around the ankle and about 22 mmHg around the knee), ankle compression socks (pressure; about 22 mmHg on the back of the foot to 25 mmHg in the line from the heel to the instep of the foot), thigh support garments (pressure; about 17 mmHg around the knee to about 10 mmHg around the thigh), PRO knee compressive support garments (pressure; about 18–20 mmHg). The CG pressure gradients were provided by the manufacturer. If athletes reported that they used any other than the aforementioned CGs they were excluded from the analyses.

Furthermore, the athletes were asked, for which reason, and how often they used their CGs. Athletes reported their primary and, if applicable, secondary reason (athletes could select, if applicable, multiple secondary reasons) for using the CGs: 1) primary prevention [i.e. prevention of a sports injury that has not occurred]; 2) secondary prevention [i.e. prevention of recurrence of a sports injury previously experienced by the athlete]; 3) to aid post-exercise recovery [i.e. recovery from a competition or training in such a way that the body is prepared for the next session]; 4) to improve performance [i.e. improvement aimed at achieving a predetermined goal for a sport]; 5) to reduce symptoms of a current sports injury; 6) to look good; 7) no specific reason, and 8) other. If athletes answered ‘other’ they could report other primary or secondary reasons for using CGs. Athletes were also asked to report the frequency of the use of their CGs: 1) during, 2) directly after, or 3) the day after training or competition participation by using the following Likert-scale (percentage of the time they use CGs): ‘never’ (0%), ‘sometimes’ (1–35%), ‘regularly’ (36–75%), ‘often’ (76–95%), or ‘always’ (96–100%).

The athletes that aimed to prevent (re-)injury, reported their perceived effects on primary or secondary injury prevention as ‘strong’, ‘partial’, or ‘no effects’. Athletes who indicated that they used the CG to aid post-exercise recovery, to improve performance, or to reduce symptoms of a current sports injury reported the perceived effects of the indicated reason as ‘positive’, ‘neutral’, or ‘negative’. The authors decided on these descriptors in order to attain rough estimates and directions of the perceived effects.

The questionnaire was piloted before the study started. Based on the results of the pilot (unpublished) questions regarding the reasons for CGs use, sports injuries, and perceived affect were adjusted to the version used in this study. For the sections CG use and sports injuries questions were combined and wording was changed to make questions more specific. For the perceived effect section the answer categories were changed from a percentage score to the aforementioned answer categories used in this study.

### Statistical analysis

All data were analysed using SPSS (version 22, IBM, Armonk, New York, USA.). Athletes were included in the analyses if they completed the personal characteristics section of the questionnaire. Athlete characteristics are reported as means and standard deviations (SD) for continuous data, median and 25–75% interquartile range (IQR) for numerical data that were not normally distributed, and percentage and frequency for categorical data.

In order to see if there were any significant differences in the characteristics of the endurance and non-endurance athletes the Chi-squared test and the Student’s T-test were used for ordinal and continuous variables, respectively. If the continuous variables were not normally distributed, the Mann-Whitney U-test was used.

The prevalence of sports injuries was calculated as the number of reported sports injuries divided by the number of athletes at risk [23]. Additionally, the sports injury incidence rate (the number of injuries per 1000 training hours (95% interval [CI])) during the past three months was calculated as follows: (number of new sports injuries during the past three months/number of athletes at risk)*(1000/hours spent training during the past three months) [24]. For the purpose of this manuscript, only the first reported past and current sports injuries were included in the analysis.

The association between the characteristics of athletes who reported running as their primary sport and who used the PRO compression socks or tubes and the odds of a current lower leg sports injury was investigated using multivariate logistic regression analyses [25]. In order to increase the power of this analyses, only runners wearing compressions socks and tubes were included in these analyses. The following characteristics were included in the analyses: age, sex, body mass index, a lower leg sports injury during the 12 months prior to the study, type of CG used, use of > 1 CG, CG use, training parameters during the last three months, and participation in competitions in the last 12 months. Older age, female sex, a higher body mass index, a lower leg sports injury during the 12 months prior to the study, and a lower average weekly running distance (injured athletes were hypothesized to run a lower weekly distance [26]) were expected to be associated with a higher odds of a current sports injury [26, 27]. The first multivariate model was adjusted for age and sex only. Based on the first multivariate model, scientific literature, and consensus among the authors, the following variables were included in the full multivariate regression model: age, sex, lower leg sports injury during the 12 months prior to the study, use of CGs, and average running distance per week. In order to test the assumptions of multicollinearity, a tolerance of < 0.1 and variance inflation factor > 10 were used [28, 29]. A priori alpha was set at 0.05.

## Results

### Sample characteristics: which athletes use CGs

**Fig. 1:**
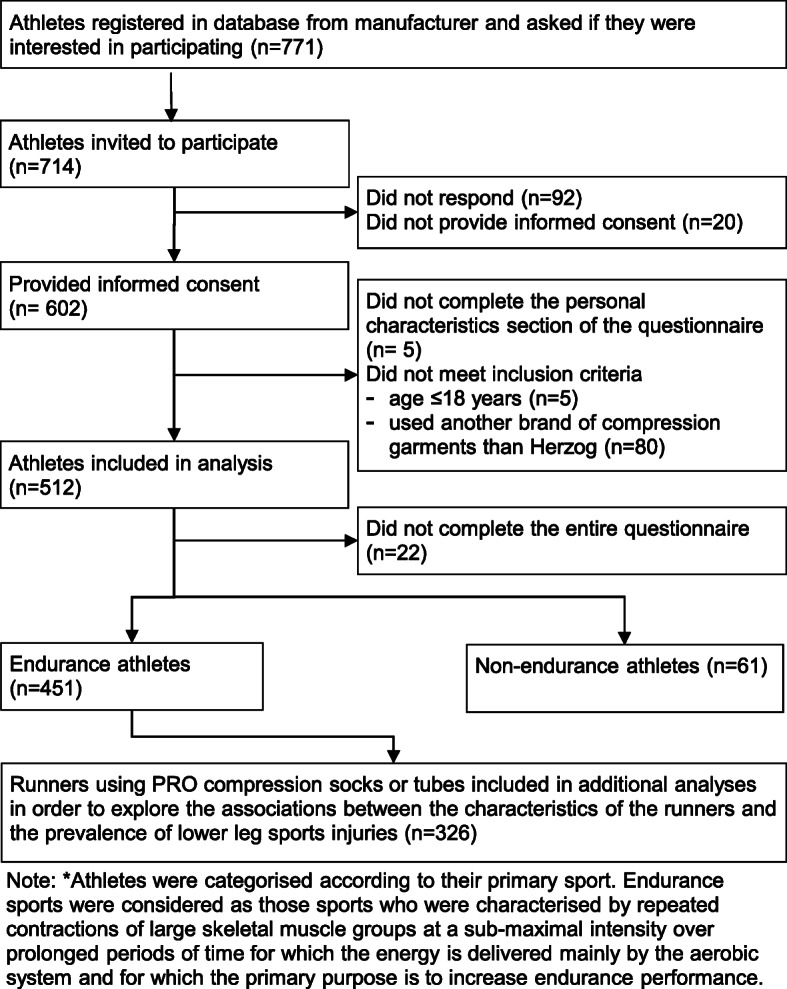
Flowchart of participants

In total, 602 of 714 invited participants provided informed consent (Fig. 1). Of these, 512 athletes completed the personal characteristics section of the questionnaire, met the inclusion criteria, and were included in this study. Fully completed questionnaires were returned by 490 (95.7%) of these athletes.

The 512 included athletes were categorized into endurance (88.1%, *n*=451) and non-endurance athletes (11.9%, *n*=61) (Table 1). Running was the most frequently reported primary sport (84.7%, *n*=382) for endurance athletes and obstacle course racing (24.6%, *n*=15) for non-endurance athletes. PRO sport compression socks were most frequently used by all athletes (59.2%, *n*=303), followed by PRO sports compression tubes (27.0%, *n*=138) .

### Why athletes use CGs

The most frequently reported primary reason of all athletes for using a CG was secondary sports injury prevention (48.6%, *n*=243) (Table 2). The second most common primary reason to use a CG was to reduce symptoms of a current sports injury (14.8%, *n*=74). The most reported secondary reason for using a CG was to facilitate post-exercise recovery (43.0%, *n*=215).

**Table 2:** Reasons for the use of compression garments (CGs)

	Endurance athletes (***n*** = 442)		Non-endurance athletes (***n*** = 58)	
Primary reason for using CGs, n (%)				
Primary prevention	60	(13.6)	6	(10.3)
Secondary prevention	211	(47.7)	32	(55.2)
Post-exercise recovery	63	(14.3)	3	(5.2)
Sports performance improvement	39	(8.8)	7	(12.1)
To reduce symptoms of a current sports injury	64	(14.5)	10	(17.2)
To look good/professional	1	(0.2)	0	(0.0)
Other	3	(0.7)	0	(0.0)
Secondary reasons for using CGs^*^, n (%)				
Primary injury prevention	141	(31.9)	21	(35.6)
Secondary injury prevention	145	(32.8)	18	(30.5)
To decrease symptoms of a current sports injury	52	(11.8)	12	(20.3)
Post-exercise recovery	184	(41.6)	31	(52.5)
Sports performance improvement	131	(29.6)	24	(40.7)
To look good/professional	22	(5.0)	1	(1.7)
Comfortable to wear	5	(1.1)	0	(0.0)
Other	3	(0.7)	0	(0.0)
Time since first use of CGs				
< 6months	43	(9.5)	5	(8.2)
6–12 months	77	(17.1)	11	(18.0)
1-2 years	113	(25.1)	15	(24.6)
2-3 years	87	(19.3)	11	(18.0)
3-4 years	62	(13.7)	9	(14.8)
4-5 years	41	(9.1)	6	(9.8)
> 5 years	28	(6.2)	4	(6.6)

^*^multiple answers possible

### When athletes use CGs

**Fig. 2:**
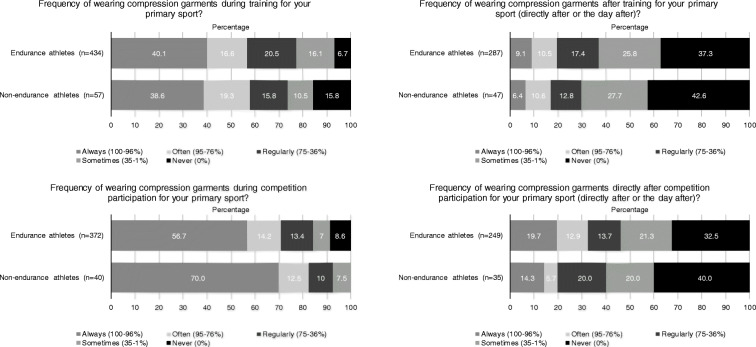
Use of compression garments during and after training or competition participation

The majority of athletes “always” (100–96% of the time) or “often” (95–75% of the time) used a CG during training (56.8%, *n*= 279) and competitions (72.9%, *n*=264) for their primary sport (Fig. 2). However, athletes only “sometimes” (35–1% of the time) or “never” (0% of the time) used their CG directly after or the day after training (64.1%, *n*=214) or competition participation (54.6%, *n*=155) for their primary sport.

### Perceived effect of CGs reported by the athletes

**Fig. 3:**
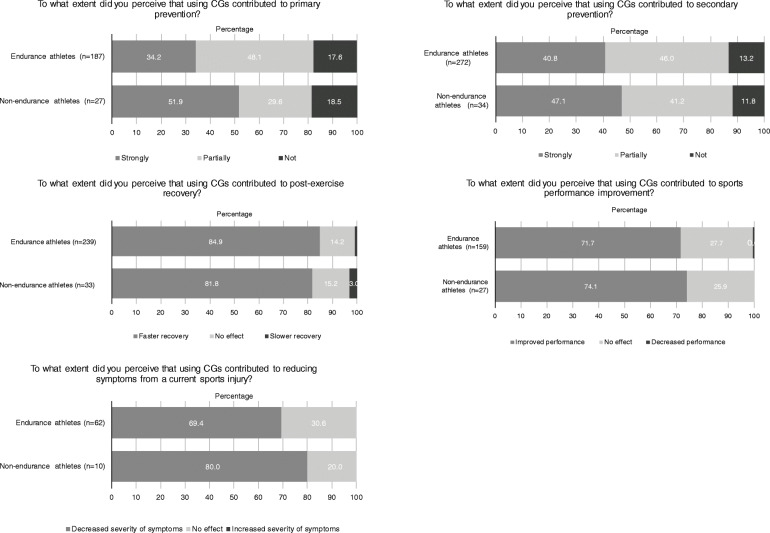
Perceived contribution of compression garments reported by the athletes on primary and secondary prevention, post-exercise recovery, sports performance improvement, and reducing symptoms of a current sports injury

Figure 3 shows the perceived benefit of the CGs reported by the athletes for the reasons for which they indicated to use their CGs, i.e. primary injury prevention, secondary injury prevention, post-exercise recovery, sports performance improvement, and symptoms of a current sports injury. Among the endurance and non-endurance athletes, 77.1% (*n*=236) and 88.2% (*n*=30) perceived that using a CG “partially” or “strongly” contributed to secondary injury prevention, respectively. Further, 84.9% (*n*=203) and 81.8% (*n*=27) perceived that they recovered faster after exercise when using CGs, respectively.

### Sports injuries

#### Past sports injuries

Of the endurance and non-endurance athletes, 84.9% (*n*=372) and 84.2% (*n*=48) reported a sports injury in the past, respectively (Table 3). Endurance athletes more often reported sports injuries with a gradual onset than non-endurance athletes (*p*=0.046). The most common injury locations reported by endurance athletes were the lower leg (64.8%, *n*=241) and knee (16.4%, *n*=61). For non-endurance athletes the most common injury locations were the lower leg (60.4%, *n*=29) and ankle (16.7%, *n*=8). Of all athletes (i.e. endurance and non-endurance athletes combined) who reported a sports injury in the past, 72.8% (*n*=306) used a CG for this injury.

**Table 3:** Past and current sports injuries

	Past sports injuries					Current sports injury				
	Endurance athletes (***n*** = 438)		Non-endurance athletes (***n*** = 61)		***P***-value	Endurance athletes (***n*** = 438)		Non-endurance athletes (***n*** = 61)		***P***-value
Sports injury, n (%)	372	(84.9)	48	(84.2)	0.886	89	(20.3)	12	(19.7)	0.897
Onset, acute, n (%)	110	(29.6)	21	(43.8)	0.046^*^	30	(33.7)	4^*^	(36.4)	0.861^†^
Onset gradual, n (%)	262	(70.4)	27	(56.3)		59	(66.3)	7^*^	(63.6)	
Use of CGs because of this sports injury, n (%)	272	(73.1)	34	(70.8)	0.738	62	(69.7)	10	(90.9)	0.139
Injury location, n (%)										
Hip	13	(3.5)	1	(2.1)		6	(7.6)	0	(0.0)	
Gluteal Muscle	0	(0.0)	0	(0.0)		0	(0.0)	0	(0.0)	
Groin	3	(0.8)	0	(0.0)		2	(2.2)	0	(0.0)	
Upper leg	0	(0.0)	1	(2.1)		0	(0.0)	0	(0.0)	
Hamstring	3	(0.8)	1	(2.1)		3	(3.4)	0	(0.0)	
Knee	61	(16.4)	5	(10.4)		4	(4.5)	1	(8.3)	
Lower leg	241	(64.8)	29	(60.4)		56	(62.9)	8	(66.7)	
Shin	92	(40.2)	9	(32.1)		20	(38.5)	4^‡^	(57.1)	
Calf	92	(40.2)	12	(42.9)		16	(30.8)	2^‡^	(28.6)	
Achilles tendon	45	(19.7)	7	(25.0)		16	(30.8)	1^‡^	(14.3)	
Ankle	26	(7.0)	8	(16.7)		9	(10.1)	2	(16.7)	
Foot/toes	21	(5.6)	3	(6.3)		9	(10.1)	0	(0.0)	
Other	4	(1.1)	0	(0.0)		0	(0.0)	1	(8.3)	
Type of injured structure, n (%)										
Muscle/tendon	280	(75.3)	35	(72.9)		64	(71.9)	8	(66.7)	
Joint	20	(5.4)	3	(6.3)		4	(4.5)	0	(0.0)	
Ligament	23	(6.2)	3	(6.3)		5	(5.6)	1	(8.3)	
Bone	26	(5.5)	3	(6.3)		4	(4.5)	1	(8.3)	
Shin splint	12	(3.2)	2	(4.2)		5	(5.6)	1	(8.3)	
Nerve	3	(0.8)	0	(0.0)		0	(0.0)	0	(0.0)	
Bursa	2	(0.5)	0	(0.0)		0	(0.0)	0	(0.0)	
Other	6	(1.6)	2	(4.2)		7	(7.9)	1	(8.3)	
Type of muscle injury, n (%)										
Strain	15	(5.4)	1	(2.9)		2	(3.1)	0	(0.0)	
Partial rupture	28	(10.0)	6	(17.1)		7	(10.8)	1	(14.3)	
Full thickness rupture	4	(1.4)	1	(2.9)		2	(3.1)	0	(0.0)	
Inflammation	55	(19.6)	4	(11.4)		8	(12.3)	0	(0.0)	
Overload injury	151	(53.9)	14	(40.0)		37	(56.9)	5	(71.4)	
Cramp	12	(4.3)	2	(5.7)		3	(3.1)	0	(0.0)	
Other	15	(5.4)	7	(20.0)		7	(10.8)	1	(14.3)	
Time since occurrence, months n (%)										
< 3	42	(11.3)	4	(8.3)		-	-	-	-	
3 to 6	47	(12.6)	5	(10.4)		-	-	-	-	
6 to 9	35	(9.4)	3	(6.3)		-	-	-	-	
9 to 12	78	(21.0)	8	(16.7)		-	-	-	-	
12 to 24	34	(9.1)	6	(12.5)		-	-	-	-	
24 to 36	50	(13.4)	6	(12.5)		-	-	-	-	
36 to 48	26	(7.0)	3	(6.3)		-	-	-	-	
48 or longer	44	(11.8)	10	(20.8)		-	-	-	-	
Unclear	16	(4.3)	3	(6.3)		-	-	-	-	
						-	-	-	-	
Duration of sports injury, months n (%)										
< 3	149	(40.1)	14	(29.2)		40	(44.9)	1^‡^	(9.1)	
3 to 6	106	(28.5)	13	(27.1)		20	(22.5)	4^‡^	(36.4)	
6 to 9	45	(12.1)	5	(10.4)		2	(2.2)	2^‡^	(18.2)	
9 to 12	37	(9.9)	8	(16.7)		7	(7.9)	0^‡^	(0.0)	
12 to 24	8	(2.2)	2	(4.2)		8	(9.0)	0^‡^	(0.0)	
24 to 36	8	(2.2)	3	(6.3)		4	(4.5)	0^‡^	(0.0)	
36 to 48	4	(1.1)	2	(4.2)		3	(3.4)	1^‡^	(9.1)	
48 or longer	7	(1.9)	0	(0.0)		4	(4.5)	2^‡^	(18.2)	
Unclear	8	(2.2)	1	(2.1)		1	(1.1)	1^‡^	(9.1)	

*Abbreviations*: CG ompression garment

^*^because of a missing value the sum of these frequencies does not correspond with the total number of sports injuries

^†^p-value indicates the results for the test between the number of sports injuries with an acute onset versus a gradual onset

^‡^because of a missing value the sum of these frequencies does not correspond with the total number of lower leg sports injuries

#### Current sports injuries

In total, 20.2% (*n*=101) of all athletes reported having a current sports injury (Table 2). The point prevalence of current sports injuries for endurance and non-endurance athletes was 20.3 and 19.7%, respectively. The sports injury incidence rate during the past three months was 2.0 (95% CI 0.7–3.3) and 0.9 (95% CI − 1.5-3.2) per 1000 training hours for endurance and non-endurance athletes, respectively. Most current sports injuries had a gradual onset. Lower leg (62.9%, *n*=56), ankle (10.1%, *n*=9), and foot/toe (10.1%, n=9) injuries were the most common in endurance athletes, and lower leg 66.7% (*n*=8) and ankle 16.7% (*n*=2) injuries in non-endurance athletes. Of all athletes reporting a current sports injury, 71.3% (*n*=72) used a CG for this injury.

#### Factors associated with a current sports injury in runners using CGs

PRO sports compression socks or tubes were used by 326 runners. These runners were included in the logistic regression analyses in order to explore the associations between the prevalence of a current lower leg sports injury and the characteristics of these runners. Table 4 shows results of the multivariate regression analyses. Multivariate regression analysis showed that “always” using a CG during competition participation and training (OR 0.32, 95% CI 0.13–0.79), and an average running distance of ≥23 km (OR 0.72, 95% CI 0.53–0.99) were significantly associated with a lower prevalence of current lower leg sports injuries.

**Table 4:** Factors associated with current lower leg sports injuries

		Model adjusted for age and sex		Multivariate regression analysis (*n*=319)	
Variable	Sample size^*^	Odds ratio	(95% CI)	Odds ratio	(95% CI)
Age, years	**–**	**–**	–	0.99	(0.95–1.02)
Female sex	321	1.37	(0.67–2.79)	1.28	(0.61–2.69)
Body mass index	321	0.95	(0.84–1.07)		
Lower leg sports injury during the 12 months prior to this study	321	1.10	(0.54–2.22)	1.12	(0.54–2.31)
Using PRO compression socks (versus tubes)	321	1.32	(0.61–2.84)		
Use of > 1 compression garment	321	1.02	(0.48–2.14)		
Use of compression garment during the last 3 months					
Always^†^ during training and competition vs ≤often^‡^ during training and competition	319	**0.31**	**(0.13–0.77)**	**0.32**	**(0.13–0.79)**
Always during training and competition vs always after training and competition.	111	0.14	(0.01–2.81)		
Training parameters during the last 3 months					
Average running distance per week ≥23 km	321	**0.43**	**(0.22–0.87)**	**0.72**	**(0.53–0.99)**
Duration of training for primary sports, hours	313	0.90	(0.75–1.20)		
Duration of training combined for primary, secondary, and tertiary sport	315	1.02	(0.94–1.20)		
Competition participation during the last 12 months					
Frequency of competition participation for primary sport	276	1.01	(0.97–1.04)		
Frequency of competition participation for primary, secondary, and tertiary sport combined	277	1.00	(0.98–1.03)		

Abbreviations; 95% CI, 95% confidence interval; km, kilometer

Note: All logistic regressions analyses were performed using athletes who reported running as their primary sport and wore compression socks or tubes; bold text denotes significant association (*p*< 0.05);

^*^Sample size only applies to the model adjusted for age and gender;

^†^always using a CG was defined as using a CG during 100–96% of the training sessions and competition;

^‡^often or less using a CG was defined as using a CG during 96% of the training sessions and competition or less

## Discussion

The aim of this study was to describe which athletes use CGs, why athletes use CGs, and when athletes use CGs. Of the total 512 participating athletes, 88.1% (*n*=451) and 11.9% (*n*=61) were endurance athletes and non-endurance athletes respectively. The most reported primary sport for the endurance athletes was running (84.7%, *n*=382). For the non-endurance athletes obstacle course racing was the most reported primary sport (24.6%, *n*=15). The PRO sports compression socks (59.2%, *n*=303) and tubes (27.0%, *n*=138) were the most-often used CGs in our study. Almost half of the endurance athletes and more than half of the non-endurance athletes indicated that the most important primary reason to wear the CGs is secondary injury prevention. About 15% and 17% of the endurance and non-endurance athletes respectively reported that the second most reported primary reason was reducing symptoms of a current sport injury. These CGs were mainly used during training and competitions and to a lesser extent directly after or the day after a competition.

The best facilitator for athletes to start with (secondary) injury prevention was sustaining an injury [30]. Athletes seem to get motivated to start using preventive measures after they have suffered from a sports injury [20]. Our study showed that athletes see CGs as a preventive measure to reduce injuries. Hypothetically wearing CGs might prevent (recurring) sports injuries. Wearing CGs reduces the oscillation of the calf muscles, specifically the medial-lateral movement and anterior-posterior movement of the calf muscles during running by ~ 13% and ~ 20% respectively [26]. This reduced muscle oscillation also occurs when wearing compression shorts during a jumping movement [31, 32]. The main muscles of the calf, the gastrocnemius and soleus muscles, store energy during the beginning of the stance phase and later in the stance phase which contribute to forward propulsion and support during running [33–35]. Consequently, this reduced oscillation of these muscles during repeated movement such as running, might lead to less fatigue, [36] and could enable the plantar flexors to better attenuate the impact of the foot on the ground during early stance phase or provide greater forward propulsion later in the stance phase. The impact of the foot on the ground can be measured using the loading rate, which is a vertical ground reaction force parameter. A higher loading rate is associated with, for example, a higher risk of a tibial stress fracture [37]. Therefore, wearing CGs could aid in attenuating the impact from foot to the ground at each heel strike by reducing the oscillation of the plantar flexor muscles during running in order to reduce the risk of a (recurring) sports injury. This hypothesized mechanism by which wearing CGs could attenuate the impact of the foot to the ground might explain why endurance athletes especially runners use CGs. However, due to the cross-sectional nature of our study we could not study causal associations. Therefore, it remains to be studied if using CGs reduces the risk of a (recurring) injury.

The most mentioned secondary reason for all athletes (47%, *n*=215) - after primary and secondary injury preventions as most reported primary reasons - to use CGs was stimulating post-exercise recovery. Multiple systematic reviews have investigated the effects of CGs on post-exercise recovery. The meta-analysis from Hill et al. [38], which included 12 studies, concluded that using CGs both during and after sports participation reduced the severity of delayed-onset muscle soreness (effect size Hedges’ G=0.40, 95% CI 0.24 to 0.58). Additionally, in the meta-analysis from Engel et al. [7] it is reported that using compression clothing, defined in this study as knee-high socks, sleeves, shorts or tights, decreased leg soreness and delayed the onset of muscle fatigue (effect size mean Hedges’ G=0.67±1.06; range − 0.44 to 3.80). Furthermore, levels of creatine kinase (CK), a marker of muscle damage, and lactate, a product of anaerobic glycolysis, were found to be lower in CG users than in CG non-users [7]. Other studies reported similar findings [6, 38]. These findings might be explained by increased venous flow velocity and lymphatic outflow due to CG use [4, 7]. An increased venous flow velocity and lymphatic outflow might aid in the clearance of CK and lactate. Alternatively, the lower levels of CK and lactate could be a consequence of the pressure exerted by the CGs on the tissue reducing the diffusion of molecules from muscle cells into the intercellular plasma and thus into the venous blood flow [6]. Moreover, irrespective of the precise mechanism, these physiological effects might explain the perceived benefits on post-exercise recovery reported in our study. As stated above, within our study the athletes who used their CG for post-exercise recovery mostly used them during sports participation rather than directly after (Fig. 2). Considering the results from the above-mentioned studies it might be advisable for athletes who use CGs for sports recovery to use their CGs both during and after sports participation.

This study also reports on the perceived benefits of CGs. Of the athletes who aimed to use CGs for secondary injury prevention (80.5%), almost 90% reported that they perceived that using CGs strongly or partly contributed to this purpose. The athletes who aimed to use CGs for recovery, over 80% perceived faster recovery. Over 70% of those indicating that they use CGs for improvement of sports performance, actually perceived sports performance improvement. Perceived effects could be the sum of any biomechanical, psychological, placebo, Hawthorne, and possibly other effects. However, because of the cross-sectional nature of our study we could not investigate the contribution of these effects. It would be of interest to take the aforementioned factors into account in future research on the effects of CGs.

Regarding the sports injuries reported within our study, the point-prevalence of current sports injuries was 20.3% and 19.7% among the endurance and non-endurance athletes, respectively. Most injuries had a gradual onset (65.3%, *n*=66) and were located in the lower leg (63.4%, *n*= 64). Lower leg injuries had a point prevalence of 12.8%, which is similar to the prevalence reported in the scientific literature. Franke et al. [19] and reported a mean prevalence of lower leg running-related injuries (RRIs) of 9.2% (95% CI 7.9–10.4%) in 161 runners preparing for a half- or full-marathon event. Hollander et al. [39], who monitored 327 runners for 13 weeks while they prepared for a half-marathon event, estimated the prevalence of lower leg pain as 12.4% (95% CI not reported). In our study CGs were mostly used for secondary injury prevention of lower leg injuries by injured athletes. Further, in the subgroup of 326 runners who used either the PRO sport compression sock or tube, using multivariate logistic regression analysis, we found that athletes who always wear their CGs during training and competition were significantly associated with a lower prevalence of a current lower leg RRI. Future research is needed to study the causal relation between the use of CGs and lower leg injury incidence.

### Limitations

Some limitations of this study should be addressed. Our study could potentially have overestimated the perceived benefits of CGs because a large proportion of the participants had used a CG for several years or because the CGs were from a single manufacturer. As described in the method section, the authors chose to include athletes from a single manufacturer to ensure that all CGs had similar weaves and pressure gradients. Furthermore, within this cross-sectional study, in which data were collected using a questionnaire, we could not measure if the pressure gradient of the CGs while being worn by the athletes matched the pressure gradient reported by the manufacturer. Variation in pressure gradients achieved could arise due to the anthropometry of the athletes. Additionally, the pressure gradient of the CGs will most likely decrease over time due to wear. For future studies we advise to measure the actual achieved pressure gradients at baseline and during follow-up.

The cross-sectional nature of the study means that it was only possible to investigate associations between the prevalence of lower leg sports injuries and the characteristics of the runners, CG usage, and average training kilometres per week. It was not possible to establish whether CG use is causally related to a lower prevalence of lower leg sports injuries.

## Conclusion

This study has shown that lower extremity CGs were mostly used by endurance athletes whose primary sport was running (84.7%). Almost half of all athletes (47.5%) reported using a CG for secondary sports injury prevention as primary reason for using CGs. The second most important reason for CG use is reduction of symptoms of a current sports injury (14.5%). The most common current sports injuries were shin (23.8%) and calf (17.8%) injuries. Almost 90% of the athletes that aimed to use CGs for secondary injury prevention, perceived a strong or partly contribution of the use of CGs to this purpose. Of those indicating that they use CGs for recovery after sports or improvement of sports performance, over 80% perceived faster recovery and over 70% perceived sports performance improvement respectively. Other primary reported aims were primary prevention (13.6%), post-exercise recovery (14.3%), sports performance improvement (8.8%), and to look good (0.2%). All athletes reported that they used the CGs more often during training or competition than after the sporting activity. In the group of runners, always wearing their compression sock or tube during training and competition was significantly associated with a lower prevalence of lower extremity sports injuries. Future research on the mechanism and effects of CGs should also focus on whether CGs can actually contribute to preventing (recurring) sports injuries in athletes.

## Data Availability

The questionnaire (made in Dutch) used and/or analysed and the datasets generated and/or analysed during the current study are not publicly available because the authors do not have permission from the participants to publicly share the data, but are available from the corresponding author on reasonable request.

## Abbreviations

CG: compression garment
CI: confidence interval
CK: creatine kinase
h: hours
IQR: 25–75% interquartile range
mmHg: millimetre of mercury
RRI: running-related injuries
SD: standard deviation
STROBE: Strengthening the Reporting of Observational Studies in Epidemiology
